# Transoral Robotic Surgery for Patients with Obstructive Sleep Apnoea: A Systematic Literature Review of Current Practices

**DOI:** 10.3390/life14121700

**Published:** 2024-12-22

**Authors:** Stavroula Mouratidou, Konstantinos Chaidas

**Affiliations:** 1 ENT Department, Guys’ and St Thomas’ NHS Foundation Trust, London SE1 9RT, UK; stavroulamouratidou@gmail.com; 2 ENT Department, University Hospital of Alexandroupolis, Democritus University of Thrace—Medical School, 68100 Alexandroupolis, Greece; 3 ENT Department, Imperial College Healthcare NHS Trust, London W2 1NY, UK

**Keywords:** transoral robotic surgery, tongue base reduction, lingual tonsillectomy, epiglottic surgery, obstructive sleep apnoea

## Abstract

Transoral robotic surgery (TORS) for tongue base reduction (TBR) and/or epiglottic surgery is an effective treatment option for selected patients with moderate to severe obstructive sleep apnoea (OSA). This systematic review aims to provide an up-to-date overview of current practices and challenges associated with TORS for OSA. PubMed and Embase databases were searched up to December 2022 following PRISMA guidelines. Primary outcome measures were surgical technique, intraoperative measures, postoperative management and complications. A total of 32 articles, including 2546 patients, met the inclusion criteria. TORS was most commonly performed as part of a multilevel surgical approach. Nasotracheal intubation was the preferred method for general anaesthesia. The surgical technique for TORS tongue base and epiglottis did not differ significantly among institutions, although some variations exist. Postoperative management varied, with most authors aiming for immediate postoperative extubation, routine postoperative ward admission and early oral intake initiation. Common postoperative complications were dysphagia and bleeding, with no reported mortality. TORS is established as a safe and feasible surgical option for selected OSA patients, addressing tongue base and/or epiglottic obstruction. However, further studies are required to determine patients’ selection criteria, preferred volume of excised tongue tissue and to assess the necessity for postoperative intensive care unit monitoring.

## 1. Introduction

Obstructive sleep apnoea (OSA) is a common sleep disorder characterised by repeated episodes of partial or complete airway collapse during sleep leading to intermittent hypoxia, disrupted sleep and daytime fatigue, thus significantly affecting patients’ quality of life. According to a recent study, approximately 1 billion people aged 30–69 years suffer from this condition worldwide [1]. Despite its benign nature, untreated OSA has been associated with a higher risk of serious cardiovascular and metabolic conditions, including hypertension, diabetes mellitus, myocardial infarction, stroke and increased mortality [2,3]. Continuous positive airway pressure (CPAP) therapy is considered as the first-line treatment in OSA, especially in moderate and severe cases, but intolerance or poor long-term compliance are frequently encountered. For that reason, targeted surgery has been explored as an alternative option aiming to improve airway patency in such patients.

Airway collapse due to tongue base hypertrophy and/or epiglottic obstruction was traditionally challenging to be addressed surgically, as its access required extensive and technically demanding procedures [4], associated with high postoperative morbidity. However, advancements in medical technologies have led to the development of less invasive approaches, such as endoscopic techniques or transoral robotic surgery (TORS), providing safer and easier access to the tongue base and supraglottic region. Since the first application for OSA patients in 2010 [5], TORS is widely used for tongue base reduction (TBR) and epiglottoplasty (EGP) in patients with moderate to severe OSA and can be offered as a standalone operation or as part of a multilevel surgical intervention.

This systematic literature review aims to provide a comprehensive analysis of current practices regarding the use of TORS in OSA, focusing on perioperative management, surgical techniques and postoperative complications, and to identify any limitations that indicate the need for further investigation.

## 2. Materials and Methods

### 2.1. Search Strategy

This systematic literature review was conducted in accordance with the Preferred Reporting Items for Systematic Reviews and Meta-Analyses (PRISMA) guidelines [6]. A search of PubMed and Embase databases for relevant articles was undertaken by two independent authors (SM and KC) up to and including December 2022. Search terms were selected to identify relevant studies examining TORS as a treatment modality for patients with OSA. Search terms included “Transoral robotic surgery” OR “tors” OR “robotic tongue base surgery” OR “robotic tongue base reduction” OR “robotic epiglottic surgery” OR “robotic epiglottoplasty” AND “Obstructive sleep apnoea” OR “osa” OR “sleep apnoea” OR “obstructive sleep apnoea syndrome” OR “osas”.

**Figure 1:**
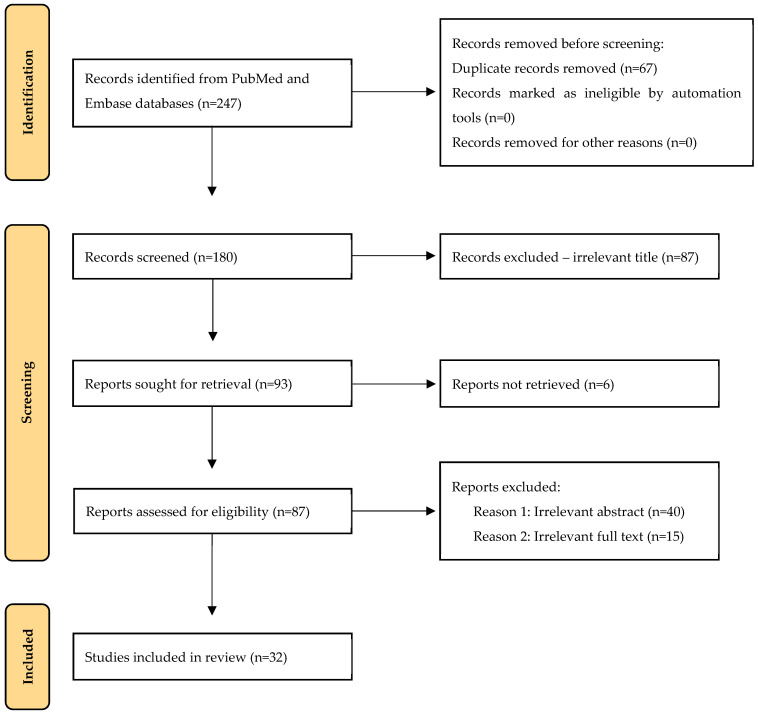
Literature search and article selection; n: number of studies.

Initially, articles were screened based on titles and abstracts for relevance, and subsequently full-text articles were evaluated for eligibility in accordance with predefined inclusion criteria. In more detail, our inclusion criteria were (1) articles published in English, (2) human studies with participants > 16 years old, (3) original research articles and (4) focusing on TORS as a treatment modality for OSA, including relevant outcome measures. Exclusion criteria were articles irrelevant to the research scope, conference abstracts, reviews, or case reports and articles not available in full text. In instances where multiple articles duplicated patient data from the same cohort, we retained the study with the largest sample for review. References of all full-text articles were manually searched, and additional relevant articles were also included (Figure 1).

The full texts were analysed and all information regarding study design, patient’s demographics, inclusion and exclusion criteria, surgical technique, intraoperative measures, postoperative management and postoperative complications were gathered. To ensure consistency, data from the identified studies were initially extracted and assessed by one investigator and subsequently verified independently by the co-author using standardised data forms.

### 2.2. Quality Assessment and Risk of Bias Assessment

**Figure 2:**
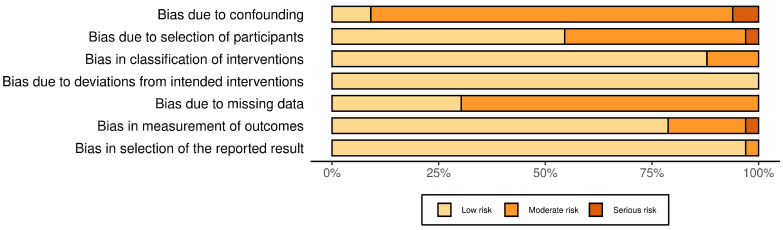
Risk of Bias assessment tool.

The Risk of Bias Assessment Tool (ROBINS-I) [7] was utilised to evaluate the quality of the included data. The risk assessment highlighted several key issues, primarily stemming from the retrospective nature of most studies. As displayed in Figure 2, concerns were predominantly related to confounding, selection criteria and missing data.

## 3. Results

Overall, 32 studies published between 2012 and 2022 were included. Eight prospective, one randomised and twenty-two retrospective studies were identified, while one remaining study provided insufficient information regarding the data collection methodology. The basic characteristics of each study are presented at Table 1 and Table 2.

### 3.1. Patients’ Characteristics

A total of 2546 patients (77.5% males) undergoing TORS for OSA were included in this review. The age ranged from 16 to 81 years, with a combined mean age of 48.7 years. The combined mean body mass index (BMI) was 29.5 kg/m^2^, ranging from 17.4 to 55.8.

Among 24 studies providing detailed inclusion criteria, the presence of moderate to severe OSA and/or CPAP intolerance or refusal were reported as primary indications for TORS in all but one study, whereas a study by Lan et al. [26] used an AHI greater than 10 episodes/hour as a cutoff value for inclusion. Several studies also included certain clinical findings for the eligibility criteria, such as evidence of obstruction at the tongue base level of flexible nasopharyngoscopy (FNE) and/or drug-induced sedation endoscopy (DISE), as well as a BMI < 35 kg/m^2^ and a Mallampati or Friedman score ≥ 3.

### 3.2. Perioperative Management

#### 3.2.1. Anaesthesia

A total of 24 studies provided adequate information regarding anaesthetic management in 1164 patients. Nasotracheal intubation was the preferred method of administering anaesthesia in the vast majority of studies, as it provides adequate space within the oral cavity and pharynx for easier robotic instruments insertion and movement. Nevertheless, orotracheal intubation is an alternative and was the method for airway management in one study [20]. Surgical tracheostomy was performed in 10.3% of cases (*n* = 120) [10,11,16,24,30,36], primarily as a prophylactic measure due to the risk of postoperative airway oedema, with decannulation occurring within the first postoperative week in most centres.

#### 3.2.2. Room Set-Up

The anaesthesia cart is positioned at the foot side of the surgical bed. The operating console is positioned away from the patient, but close enough to allow adequate communication between the primary surgeon and the rest of the theatre team [8,9,13,15,18,21,26,39]. The robotic surgical cart is most frequently placed at the patient’s right-hand side with a 45° [18] or 30° [8,21] angle to the operating table. The scrub nurse sits at the left-hand side and the assistant surgeon at the head of the operating table [18,26].

#### 3.2.3. Patient Positioning and Preparation

The patient is placed in supine position with a shoulder roll to facilitate neck extension [8,18,35]. Eye protection and a tooth guard or gauze for tongue and teeth protection can be applied. There is no need for additional preparation of the surgical field, although one study [8] described oral cavity preparation with rinsing of 0.12% chlorhexidine gluconate and local injection of epinephrine (1:100,000) and bupivacaine (2 mL) into the tongue base.

#### 3.2.4. Surgical Set-Up

A mouth gag is used to open the patient’s mouth, and a proper size tongue blade is inserted to expose the oral cavity and the tongue base. Some surgeons also use one or two silk traction sutures, placed in the anterior aspect of the tongue, to achieve better retraction and obtain adequate exposure of the tongue base [8,15,29,39]. Once appropriate exposure is achieved, three robotic arms are positioned inside the oral cavity with a 45° angle to the tongue base plane [15,18,39]. Typically, the use of a 30° up 3D camera endoscope allows better visualisation, although 0-degree 3D cameras were also used by some surgeons [9,10,15,28]. The endoscope is placed centrally and is connected with the medial robotic arm. Maryland forceps and a monopolar cautery or laser fibre are the most frequently used instruments, which are mounted on either side of the endoscope. Volner et al. [38] implemented a 1.02 mm thick silicone sheet, which was placed between the epiglottis and the tongue base to avoid thermal injury of the epiglottis during tongue base cauterisation, which can potentially cause significant scarring and prolonged dysphagia.

#### 3.2.5. Surgical Technique

- Tongue Base Reduction (TBR)

Tongue base reduction is a term referring to a tongue base operation aiming to address obstruction at this level. TORS-TBR can be performed as a midline posterior glossectomy, lingual tonsillectomy or most commonly as a combination of both procedures. Significant anatomic landmarks in order to obtain adequate exposure of the tongue base and proper orientation include the circumvallate papillae, the foramen cecum, the vallecula and the superior tip of the epiglottis and should be identified prior to dissection.

With regard to lingual tonsillectomy, the amount of removed tissue mainly relies on the surgeon’s experience and discretion, aiming to alleviate tongue base obstruction and achieve adequate airway patency [15,18]. The suggested plane of dissection is between the intrinsic tongue muscles and the hypertrophied lingual tonsils [15,28,36], whereas according to some authors, the muscle tissue can also be excised, if clinically appropriate [16,29]. The lingual tissue can be removed “en-block” or in a piecemeal way [9,31].

For the midline posterior glossectomy, the dissection commences from the foramen cecum, extending vertically to the vallecula, with attention to avoid injury of the epiglottic mucosa. It is also important to preserve nearby neurovascular structures such as the lingual and hypoglossal nerve as well as the lingual artery, as its dorsal branch can be encountered submucosally near the vallecula. The free edge of the tongue is grasped with Maryland forceps and the incision is extended laterally, thus additional tongue tissue is excised [12,13]. The extent of the tongue base musculature removal varies based on the surgeon’s discretion, but typically involves tissue resection 1.5 cm posteriorly to the foramen cecum, 3 cm in width (1–1.5 cm from the tongue midline on each side), with a depth of 1.5–2 cm from the tongue base to the epiglottis [18,20,34].

Depending on the surgeon’s preference, dissection can be performed using a monopolar cautery (30J) [12,21], thulium laser (2013 nm/15 W) or CO_2_ laser (320 μm/14 W for dissection and 7 W for coagulation) [12,18,21]. Karaman et al. [21] reported favourable outcomes using a CO_2_ laser compared to electrocautery in such procedures with less intraoperative bleeding, decreased console time and improved postoperative parameters.

- Epiglottic surgery

Partial epiglottidectomy or epiglottoplasty or epiglottoplexy are all terms used interchangeably to describe similar surgical techniques aiming to alleviate epiglottic obstruction. This procedure is performed either as a single modality or as an additional procedure during TORS in addition to tongue base reduction, especially when the epiglottis is significantly retroflexed [12,13,18,30,37,39]. The epiglottis is grasped with a Maryland dissector and the upper half or upper one-third of the epiglottis is excised. This can be done either via a wedge-shaped excision [18] or, more frequently, via a vertical division along the midline, followed by horizontal cuts in right and left lateral planes [12,13,39]. A monopolar diathermy or laser can be used for dissection and haemostasis, based on surgeons’ preference.

#### 3.2.6. Surgical Instruments

Τhe most commonly used instruments during a TORS procedure in OSA patients are the following:

- Mouth gag/retractor: Feyh–Kastenbauer retractor (most commonly used), Davis-Meyer, Boyle-Davis, Crow–Davis, Mclvor;
- Endoscope: 0- or 30-degree 3D;
- Suction: Yankauer;
- Robotic instruments: Monopolar cautery, Maryland dissector forceps (mainly 5 mm, rarely 8 mm);
- Laser: CO_2_ or thulium.

### 3.3. Intraoperative Outcome Measures

#### 3.3.1. Operating Time

The combined mean robotic set-up time was 20.7 min (range 10–70), whereas the mean robotic console operating time regarding TORS TBR and/or epiglottic surgery was 32.5 min (range 14–90). As expected, the total duration of surgery, including concurrently performed procedures such as other upper airway intervention and/or tracheostomy, was significantly longer, with a mean time of 78.6 min (range 14–246 min).

#### 3.3.2. Intraoperative Blood Loss

No case of major intraoperative haemorrhage was noted. All but one study reported that intraoperative blood loss did not exceed 30 mL, with a combined mean value of 22 mL. However, a study by Hoff et al. [11] reported that even though the mean blood loss was only 27.5 mL, a wide range from 0 to 400 mL was noted. Notably, Chang et al. [35] stated that, in the subgroup without the use of intraoperative ultrasound, two cases had significant intraoperative bleeding, which required additional surgical intervention, without specifically stating whether this was conversion to open surgery or not. Haemostatic agents were routinely used at the end of the procedure by a few surgeons [11,17].

#### 3.3.3. Volume of Excised Tissue

The mean volume of resected tongue base tissue was 9.6 mL (range 1.5–57 mL). A minimum excision of 7 mL of lingual tonsil tissue was suggested as effective, whereas removal of >50 mL was discouraged due to a potentially higher risk of complications such as neurovascular injury, without improving the surgical outcome [16,21]. In the TORS standalone group, two studies provided sufficient data, covering 19.1% of the patients in this group, with a reported mean excised volume of 27.6 mL (range 4–57 mL). Regarding the multilevel surgery group, data from eight studies, accounting for 36.5% of the patients, reported a mean excised volume of 9.3 mL (range 0.9–30 mL). Most studies measured the volume of excised tissue in ml, whereas one study [31] used mm to measure the postoperative retroglossal airway diameter.

#### 3.3.4. Additional Procedures

TORS for tongue base reduction and/or epiglottoplasty was performed and assessed as a standalone procedure for OSA patients in only nine studies (Table 1 and Table 2). The outcomes of the remaining publications refer to TORS at the tongue base ± epiglottis as part of a single multilevel operation. Among the concurrently performed procedures were nasal surgery (septoplasty, turbinoplasty or endoscopic sinus surgery), oropharyngeal surgery (palatine tonsillectomy, uvulopalatopharyngoplasty, expansion sphincter or relocation pharyngoplasty, barbed repositioning pharyngoplasty, Z-palatoplasty, lateral pharyngoplasty or anterior palatoplasty), adenoidectomy and maxillomandibular advancement surgery.

### 3.4. Postoperative Management

#### 3.4.1. Hospital Stay

Postoperatively, only 12.7% of patients required close monitoring in either intensive (ICU) or high-dependency (HDU) care units overnight or for the first 24 h, with rare exceptions requiring longer postoperative monitoring not exceeding 3 days. Some centres routinely kept patients intubated in the ICU for the first postoperative day [10,12,13,14,19,32,39], while the remaining institutions aimed for immediate postoperative extubation, unless significant airway oedema was evident. Overall, the vast majority of patients were discharged within 3 days after surgery, with a mean hospital stay of 3.8 days (range of 1–19 days).

#### 3.4.2. Diet

The time of oral feeding initiation after surgery varies among institutions, but typically occurs at the early postoperative period with a mean of 2.1 days (range of 0.2–12 days). Quick progression to oral intake even a few hours after surgery is considered reasonable, without increasing the risk of postoperative complications [14,15]. Most authors did not routinely place a nasogastric tube (NGT) postoperatively, with the exception of two studies. Namely, Karaman et al. [21] routinely inserted NGT as a precaution, even though oral feeding was commenced 6–10 h postoperatively, and NGT was removed once the patients progressed to an oral diet. Likewise, Dallan et al. [30] placed NGT in all cases, which was kept for a mean duration of 12 ± 5 days.

#### 3.4.3. Postoperative Medications

Nine studies provided data regarding the postoperative medication regime, which mainly included corticosteroids, antibiotics, analgesia, mouth gargles and gastroprotective agents [10,11,13,14,15,18,24,29,32,36]. Notably, only a few studies provided specific information about the dosage, duration and route of administration. In more detail, a 5-day course of oral amoxicillin–clavulanic acid was the most often prescribed antibiotic regime [13,14,15,18], whereas induction antibiotics were not stated in any of the included studies. After surgery, corticosteroids (dexamethasone or methylprednisolone) were administered either routinely or based on clinical features—mostly in the presence of airway oedema—twice or three times daily, with only one study specifying the duration of the course, suggesting routine prescription of steroids for 5 days [18]. The analgesia regime included paracetamol, ibuprofen and codeine or other opioids on either a regular or as required basis. All medications were initially commenced intravenously for the first postoperative days and were then switched to orals, when feasible.

### 3.5. Surgical Complications

Table 3 shows an overview of reported postoperative complications. Interestingly, the data from most studies refer to TORS as part of a multilevel surgical procedure, thus significantly affecting the reported rates of surgery-related complications.

In a total of 2494 patients, bleeding was reported in 77 cases, accounting for 3.1%. This complication was self-limited or conservatively managed in 32 of those patients (41.6%). The bleeding source was located at the tongue base in 51 cases (66.2%), whereas in 26 patients (33.8%) undergoing a multilevel surgical approach, the bleeding source was not stated. Postoperative bleeding requiring management in the operating theatre was reported in one patient (6.7%) in the TORS standalone group and in 39 cases (62.9%) in the multilevel group.

Overall, zero mortality and hypoglossal nerve injury rates were reported. Issues with swallowing dysfunction were reported in 131 cases (5.6%). Nasogastric tube placement was required in five cases, whereas there was one patient with gastrostomy tube dependence for a period of 4 months with a return to oral intake afterwards [12]. Surgical removal of oropharyngeal scarring was performed in one case [10].

Other reported complications included upper airway oedema (4.1%), taste disturbances/dysgeusia (3.8%), cardiac and pulmonary complications (>4.1%), wound infection (0.04%) and dental injury (0.04%). Postoperative pain and dehydration were also commonly encountered, leading to readmission in 42 cases (2.2%).

## 4. Discussion

Transoral robotic surgery (TORS) in the management of refractory OSA patients has gained worldwide acceptance. Previous systematic reviews and meta-analyses have mainly focused on the efficacy of TORS in select patients by evaluating relevant subjective and objective parameters [40,41,42,43]. In contrast, this systematic review provides an overview of current clinical practice for patients undergoing TORS across various institutions by focusing on potential differences in perioperative management including patient preparation, surgical technique and postoperative management. Moreover, this study evaluated the safety of the procedure by summarising reported complications. Overall, despite the remarkable heterogeneity among the included studies, TORS is considered as a safe procedure with low morbidity rates.

In the vast majority of studies, eligible patients for TORS were those with moderate to severe OSA, CPAP intolerance or refusal, and the presence of obstruction at the level of the tongue base and/or epiglottis on clinical examination. Transoral robotic surgery is performed under general anaesthesia typically administered via nasotracheal intubation, as this approach offers a wider surgical field. Although initial studies recommended routine tracheostomy for TORS patients, primarily as a precautionary measure [6,16,24,30,36], current practice in most institutions has now changed, and recent data indicate that there is no need for routine tracheostomy in OSA patients undergoing TORS [8]. In fact, avoiding tracheostomy in such patients is associated with decreased surgical time, shorter hospital stays and lower aspiration rates, compared to those undergoing tracheostomy [36]. Of course, prophylactic tracheostomy may still be required when TORS is performed in combination with other extensive procedures that increase the risk of postoperative airway oedema, such as MMA [30].

The choice of surgical technique for each patient should be personalised and guided by both clinical examination, including DISE, and intraoperative findings. Lingual tonsillectomy, midline glossectomy and epiglottoplasty are all feasible options and can be performed separately or in combination. TORS is an effective therapeutic modality not only in the presence of tongue base obstruction, but also for managing epiglottic collapse [44]. Interestingly, Stehan et al. [45] performed TORS epiglottoplasty as a standalone procedure in two OSA patients with favourable outcomes. While TORS can be performed as a primary procedure for a few carefully selected OSA cases, it is also often used as a “salvage” procedure or following previous unsuccessful operations [16,18,25]. It is evident that careful patient selection for TORS is crucial in order to maximise its effectiveness and minimise complication rates. Overall, a standardised algorithmic approach for surgical management in OSA is recommended, aiding clinical decision making.

Evaluating intraoperative outcome measures is essential for advancing surgical technique and optimising patient outcomes, yet few studies provided sufficient data. Assessing robotic surgical time proved challenging, as the majority of studies involved multilevel surgical procedures and frequently omitted detailed data on the duration of each separate procedure performed. However, both the robotic set-up and console time can be significantly improved as surgeons and theatre staff gain experience, indicating a learning curve that affects outcomes [8,13]. Additionally, although studies reported the volume of excised tongue tissue, only a few correlated this with surgical success. While some studies concluded that the larger volume of excised tissue was statistically associated with improved surgical success rates [15,20,31], there was no consensus on the optimal volume, and excision was often left to the surgeon’s discretion. Some authors suggested that a resected tissue volume greater than 7 mL results in a better surgical outcome [16,21], whereas others found no correlation between the volume of excised tissue and postoperative AHI reduction [32,46]. These controversies underline the necessity of establishing a personalised surgical approach based on pre- and intra-operative clinical findings.

No consensus exists regarding patients’ management during the immediate postoperative period after TORS. Some institutions suggest close postoperative monitoring in a high-dependency unit (HDU), whereas in other centres, patients remain intubated for 24 h as a precautionary measure. Overall, only a small percentage of patients (12.7%) required admission to the ICU, mainly as a precaution for those considered more prone to develop postoperative upper airway oedema [14]. The decision primarily relies on the surgeon’s experience, as well as the hospital protocols and setting. Compared to less invasive techniques addressing tongue base hypertrophy such as radiofrequency ablation, TORS patients require a prolonged hospital stay [22,23,26,33]. Nevertheless, based on the rapidly increasing level of experience gained over recent years, most authors are in favour of no ICU monitoring and aim for early discharge.

Likewise, there is no need for delay in the progression with an oral diet or routine nasogastric tube placement [14]. According to a study with one of the largest sample sizes by Vicini et al. [16], over 90% of patients were able to swallow within 48 h postoperatively. In contrast, only one study [30] reported routine NG tube placement. Speech and language therapists can be engaged in the proper evaluation of swallowing prior to progression to an oral diet [14].

Our review revealed a variation in the prevalence of the reported complications between studies, which can be attributed to discrepancies in the complication definitions and the follow-up duration. Major complications were rarely encountered, with common TORS-related postoperative complications including swallowing difficulties, taste disturbance, airway oedema and bleeding. Dysphagia, odynophagia and globus sensation were among the most often reported swallowing complications and were usually temporary. Implementation of pre- and postoperative questionnaires, such as the Swallowing Disturbances Questionnaire (SDQ) or Dysphagia Handicap Index could facilitate swallowing evaluation and early management as well as consideration for a SALT referral in cases with long-term swallowing dysfunction [12,28,29]. Taste disturbances are known to be expected following any type of oral surgery, including tonsillectomy, and are considered temporary [47]. Additionally, while bleeding is a feared complication of TORS, it fortunately seems to be relatively rare. Based on our analysis, haemorrhage occurred in approximately 3.1% of cases overall, with about half of them necessitating re-intervention in the operating room. Interestingly, bleeding was more frequent in the TORS standalone group, occurring three times more often than in the multilevel surgery group, although it was less severe as it rarely required surgical management (0.7%). The higher incidence may be attributed to the extent of surgery in the standalone group, which involved more aggressive tongue base excision compared to the multilevel group (mean volume of excised tissue: 27.6 mL vs. 9.3 mL, respectively). Overall, readmissions were most frequently due to bleeding, pain and dehydration [12].

TORS has been recognised as an effective and safe modality aiming to address tongue base and/or epiglottic obstruction in OSA patients. A recent meta-analysis concluded that TORS outcomes are similar to alternative procedures, but TORS offers greater surgical precision [48]. While major or life-threatening complications are rare in TORS, some studies indicate potentially higher morbidity rates compared to less invasive surgical approaches. Aynaci et al. compared Radiofrequency Tongue Ablation (RFTA) with TORS [23] and found that AHI, minimum arterial oxygen saturation and ESS scores were significantly improved postoperatively in the TORS group, with similar complication rates but a longer operative time and hospital stay compared to the RFTA group. When compared to endoscopic coblation tongue base reduction, TORS generally allows for a wider surgical field and more accurate resection of tongue base tissue, although findings on complications and success rates vary [20,22,26,29,49]. On the other hand, although upper airway stimulation (UAS) is not considered as a substitute for TORS, it shows benefits for patients with tongue base obstruction due to relaxed muscle tone, offering fewer complications and faster recovery [25,37]. In fact, one study found that TORS complication rates were significantly higher (20%) when compared to a UAS subgroup (2%) [37]. Overall, each technique has unique advantages and disadvantages, while TORS is a safe and effective surgical approach, offering the advantage of precision and wider tongue base tissue resection.

It is important to note that airway obstruction in adult patients with OSA is usually multilevel, with several factors playing a role. For that reason, surgical management of OSA is challenging, requiring a personalised approach instead of a traditional ‘one size fits all approach’. This has led to the establishment of a wide range of surgical approaches, with most published studies utilising multilevel approaches combining different techniques for nasal and/or pharyngeal surgery, including TORS. This diversity poses significant challenges in isolating the complications and effectiveness of TORS. To address these challenges, this review assessed data for TORS separately wherever possible, despite the predominance of multilevel approaches, providing meaningful comparisons when feasible.

Overall, this review aims to provide valuable insights regarding current practices in TORS for OSA patients; however, certain limitations need to be acknowledged. First, most included studies were retrospective, with relatively small sample sizes and heterogenous and low-quality methodology; hence, the interpretation of the results should be made with caution. Furthermore, patient selection bias should be addressed, as a variation in patients’ BMI and comorbidities were noticed, which can potentially affect outcomes. In fact, four studies [9,10,11,16] included patients with a BMI > 40 kg/m^2^. Additionally, the variability in surgical techniques and the lack of standardisation challenge the consistency and comparability of outcomes. Finally, limitations in analysing postoperative complications exist, mainly due to the lack of a standardised definition and classification system and due to TORS commonly being performed concurrently with other procedures, making it difficult to isolate TORS-specific complications.

## 5. Conclusions

In summary, this study provides a comprehensive overview of the current practice for TORS in OSA patients, offering valuable insights into patients’ preparation, surgical techniques, postoperative management and complications. Overall, TORS represents a safe and feasible option for refractory OSA patients with tongue base and/or epiglottic obstruction, either as a standalone procedure or as part of a multilevel operation. It offers surgical precision, 3D visualisation and a wider surgical field compared to other techniques for tongue base reduction. Nasotracheal intubation is the preferred method for airway management, while early initiation of an oral diet, facilitating early discharge without impacting complication rates, was the goal of most recent studies. Considering some differences in performed surgical technique and perioperative management, further studies are required, especially in order to determine the optimal volume of excised tongue tissue and assess the necessity for postoperative ICU monitoring.

## Figures and Tables

**Table 1:** Individual study characteristics.

Study	Year	Design	Number of Patients (n)	TORS Procedures		Additional Procedures (Including Other Robotically Assisted Procedures) (n/N, %)	Follow-Up (Months)
				Tongue Base Reduction (n/N, %)	Epiglottis (n/N, %)		
Friedman [8]	2012	historical cohort	27	Midline glossectomy (27/27, 100%)	-	Modified ZPP (27/27, 100%)	>6
Lee [9]	2012	prospective, non- randomised trial with historical controls	20	LT (20/20, 100%)	-	UPPP (20/20, 100%)	>3 (range 4–19)
Lin [10]	2013	retrospective case series	12	Midline glossectomy (12/12, 100%)	-	None	>6
Hoff [11]	2014	multicentre, single-arm, retrospective case series	293	LT ± partial glossectomy (293/293, 100%)	±epiglottectomy (27/293, 9.2%) or EGP (22/293,7.5%)	Robotic palatine tonsillectomy (10/293, 3.4%), Other robotic procedure (5/293, 1.7%), UPPP (194/293, 66.2%), Lateral pharyngoplasty (36/293, 12.3%), Palatine tonsillectomy (94/293, 32.1%), Palatal Z-plasty (28/293, 9.6%)	3
Glazer [12]	2014	retrospective cohort	166	LT (166/166, 100%) + partial midline glossectomy (65/166, 39.2%)	±epiglottectomy (79/166, 47.6%)	All patients underwent concurrent procedures including: Tonsillectomy (70/166, 42.2%), UPPP (47/166, 28.3%), Palatoplasty (47/166, 28.3%), Pharyngoplasty (42/166, 25.3%), Turbinate reduction (36/166, 21.7%), Uvulectomy (25/166, 15.1%), Septoplasty (3/166, 1.8%), Adenoidectomy (1/166, 0.6%)	≥3
Toh [13]	2014	retrospective review of prospective collected data	40	LT (40/40, 100%) ± midline glossectomy	partial epiglottectomy (40/40, 100%)	+primary tonsillectomy with uvulopalatal flap (40/40, 100%)	>6
Richmon [14]	2014	retrospective	7	LT (7/7, 100%)	-	N/A	<1 (10 days)
Muderris [15]	2014	retrospective	6	LT (6/6, 100%)	±EGP (1/6, 16.7%)	None	>3
Vicini [16]	2014	multicentre observational retrospective	243	LT (243/243, 100%) ± midline glossectomy -standalone (34/243, 14%)	±SGP	part of multilevel surgery: ±palatal: tonsillectomy (146/243, 60%), UPPP (146/243, 60%), nasal surgery (143/243, 59%), other palate surgery: Pang–Tucker Woodson expansion sphincter pharyngoplasty, palatal Z-plasty.	>3
Thaler [17]	2015	prospective, nonrandomised trial with historical controls	75	posterior glossectomy and limited lateral pharyngectomy (75/75, 100%)	-	+UPPP (75/75, 100%)	>3
Arora [18]	2016	prospective case series	14	LT + midline glossectomy (14/14, 100%)	±wedge EGP (10/14, 71.4%)	None	12–24
Kayhan [19]	2016	retrospective	25	LT + midline glossectomy (25/25, 100%)	±EGP (24/25, 96%)	±UPPP (22/25, 88%), anterior palatoplasty (22/25, 88%), tonsillectomy (18/25, 72%), pharyngoplasty (18/25, 72%)	3
Folk [20]	2017	retrospective	45	LT ± midline glossectomy (45/45, 100%) –performed as standalone (6/45, 13.3%)	-	+additional procedures (39/45, 87%) including turbinate reduction, tonsillectomy, septoplasty, UPPP	>1
Karaman [21]	2017	prospective	20	LT + midline glossectomy (20/20, 100%)	-	N/A	Mean 12 (sleep study at 6 months)
Hwang [22]	2018	retrospective case-control	16	LT + midline glossectomy (16/16, 100%)	-	+lateral pharyngoplasty and tonsillectomy (16/16, 100%)	>6
Aynaci [23]	2018	prospective	20	TBR (20/20, 100%)	-	+anterior palatoplasty and uvulectomy (20/20, 100%) ±tonsillectomy	N/A
Bonnecaze [24]	2018	prospective—single centre	8	LT ± midline glossectomy (8/8, 100%)	-	±Phrayngoplasty (4/8, 50%), tonsillectomy (1/8, 12.5%)	6
Huntley [25]	2018	retrospective	24	TBR (24/24, 100%)	-	N/A	>1
Lan [26]	2019	retrospective	16	LT + midline glossectomy (16/16, 100%)	-	+UPPP (16/16, 100%)	>3
Turhan [27]	2019	prospective (single-arm, observational cohort)	64	LT ± midline glossectomy (64/64, 100%)	±EGP (7/64, 10.9%)	±UPPP (42/64, 65.6%), ESP (15/64, 23.4%)	>3
Paker [28]	2019	retrospective and prospective cohort	39	TBR (39/39, 100%) As standalone (7/39, 17.9%)	-	±TORS UPPP (31/39, 79.5%), TORS tonsillectomy (12/39, 30.8%)	>11
Babademez [29]	2019	prospective	37	LT + midline glossectomy (37/37, 100%)	-	None	6
Dallan [30]	2019	retrospective cohort	3	TBR (3/3, 100%)	-	+MMA (3/3, 100%)	N/A
Cho [31]	2019	retrospective	16	LT ± midline glossectomy (16/16, 100%)	-	+palatal surgery (ESP or relocation pharyngoplasty) and septoturbinoplasty and tonsillectomy (16/16, 100%)	3
Cambi [32]	2019	retrospective	20	LT (20/20, 100%)	±EGP	+ESP and septoplasty (20/20, 100%)	6
Kim [33]	2020	retrospective cohort	1016	tongue resection (1016/1016, 100%)	±epiglottis surgery (164/1016, 16.2%)	±palatal surgery (797/1016, 78%), nasal surgery (151/1016, 15%), other hypopharyngeal surgery apart from epiglottis (9/1016, 0.9%)	N/A
Tsou [34]	2021	retrospective case series	62	LT + midline glossectomy (62/62, 100%)	-	Group1: + BRP (31/62, 50%) Group2: + UPPP (31/62, 50%)	1
Chang [35]	2021	cohort study with historical control	93	LT + midline glossectomy (93/93, 100%)	-	+TORS UPPP (93/93, 100%)	6
Chekkoury Idrissi [36]	2021	randomised	20	LT (20/20, 100%)	-	±tracheostomy (10/20, 50%)	12
Stewart [37]	2021	retrospective cohort	35	LT + midline glossectomy (35/35, 100%)	+partial epiglottectomy (35/35, 100%)	None	N/A
Volner [38]	2021	N/A	7	TBR (7/7, 100%)	-	±palatine tonsillectomy	N/A
Baptista [39]	2022	retrospective	57	partial midline glossectomy + LT (57/57, 100%)	±EGP (28/57, 49%)	all as single multilevel surgery: ±Pharyngoplasty (94%), tonsillectomy (66%), septoplasty (58%), turbinoplasty (56%), adenoidectomy (5%), nasal endoscopic surgery (3%)	>6

Abbreviations: TORS = transoral robotic surgery, RFTA = radiofrequency tissue ablation, SMILE = submucosal coblation, vs = versus, LT = lingual tonsillectomy, BOT = base of tongue, UPPP = uvulopalatopharyngoplasty, ESP = expansion sphincter pharyngoplasty, BRP = barbed repositioning pharyngoplasty, TBR = tongue base reduction, SGP = supraglottoplasty, EGP = epiglottoplasty, TBS = tongue base suspension, ZPP = Z-palatoplasty, MMA = maxillomandibular advancement, (+) = all patients had the procedure, (±) = some of the patients had the procedure (if (N) available stated), N/A = information not available.

**Table 2:** Outcome measures provided by each study.

Study	Number of Patients (n)	Surgical Approach		Intraoperative Data	Postoperative Management	Surgical Complications	
		Single Level	Multilevel			Bleeding Other	
Friedman [8]	27		+	+	+	+	+
Lee [9]	20		+	+	+	+	+
Lin [10]	12	+		+	+	+	+
Hoff [11]	293	+	+	+	-	+	+
Glazer [12]	166		+	+	+	+	+
Toh [13]	40		+	+	+	+	+
Richmon [14]	7	+		-	+	-	-
Muderris [15]	6	+		+	+	+	+
Vicini [16]	243	+	+	+	+	+	+
Thaler [17]	75		+	-	-	+	+
Arora [18]	14	+		+	+	+	+
Kayhan [19]	25	+	+	+	+	+	-
Folk [20]	45	+	+	+	+	-	-
Karaman [21]	20	+		+	+	+	-
Hwang [22]	16		+	+	+	+	+
Aynaci [23]	20		+	+	+	+	-
Bonnecaze [24]	8	+	+	+	+	+	+
Huntley [25]	24	+		+	+	+	+
Lan [26]	16		+	-	+	+	+
Turhan [27]	64	+	+	+	+	+	+
Paker [28]	39	+	+	+	+	+	+
Babademez [29]	37	+		+	+	+	+
Dallan [30]	3		+	-	+	+	+
Cho [31]	16		+	+	+	+	+
Cambi [32]	20		+	+	+	+	+
Kim [33]	1016	+	+	-	+	+	+
Tsou [34]	62		+	+	+	+	+
Chang [35]	93		+	+	-	+	+
Chekkoury Idrissi [36]	20	+	+	+	+	+	+
Stewart [37]	35	+		-	+	+	+
Volner [38]	7	+	+	-	-	+	+
Baptista [39]	57		+	+	+	+	+

**Table 3:** Overview of postoperative complications.

Complications	Standalone TORS, n/N (%)	Multilevel, n/N (%)	Overall, n/N (%)
**Bleeding**	**15/168 (8.9%)**	**62/2326 (2.6%)**	**77/2494 (3.1%)**
Requiring intervention ^	1/168 (0.6%)	39/2326 (1.7%)	40/2494 (1.6%)
Self-limited/medical treatment/transient ^^	12/168 (7.1%)	20/2326 (0.8%)	32/2494 (1.2%)
Unspecified	2/168 (1.2%)	3/2326 (0.1%)	5/2494 (0.2%)
Airway oedema *	**10/148 (6.8%)**	**85/2188 (3.9%)**	**95/2336 (4.1%)**
Requiring intervention ^	-	3/2188 (0.1%)	3/2336 (0.1%)
Self-limited/medical treatment/transient ^^	10/148 (6.8%)	73/2188 (3.3%)	83/2336 (3.6%)
Unspecified	-	9/2188 (0.4%)	9/2336 (0.4%)
Swallowing **	**17/148 (11.5%)**	**114/2188 (5.2%)**	**131/2336 (5.6%)**
Requiring intervention ^	4/148 (2.7%)	2/2188 (0.1%)	6/2336 (0.3%)
Self-limited/medical treatment/transient ^^	13/148 (8.8%)	104/2188 (4.8%)	117/2336 (5%)
Unspecified	-	8/2188 (0.4%)	8/2336 (0.3%)
Taste ***	**26/148 (17.6%)**	**63/2188 (2.9%)**	**89/2336 (3.8%)**
Requiring intervention ^	-	-	
Self-limited/medical treatment/transient ^^	26/148 (17.6%)	55/2188 (2.5%)	89/2336 (3.8%)
Unspecified	-	8/2188 (0.4%)	
Cardiopulmonary (MC pneumonia) ****	**-**	**>90/2188 (>4.1%)**	**>90/2188 (>4.1%)**
Requiring intervention ^	-	-	
Self-limited/medical treatment/transient ^^	-	>90/2188 (>4.1%)	>90/2188 (>4.1%)
Unspecified	-	-	
**Readmissions due to pain/dehydration**	**8/148 (5.4%)**	**44/2188 (2%)**	**52/2336 (2.2%)**
Requiring intervention ^	-	-	
Self-limited/medical treatment/transient ^^	8/148 (5.4%)	44/2188 (2%)	52/2336 (2.2%)
Unspecified	-	-	

* Lingual and/or pharyngeal oedema; ** Dysphagia/odynophagia, globus, impaired swallowing; *** Dysgeusia, taste disturbances; **** Acute cardiac event, PE, pneumonia, other cardiopulmonary complications, interventions in detail: return to OR for bleeding, prolonged intubation for airway, NGT/gastrostomy or surgical removal of oropharyngeal scarring for swallowing; ^ Surgical management ^^ Transient was commonly not clearly defined regarding duration and was used to address resolution of symptoms eventually.

## Data Availability

Data were gathered in pre-formed Excel spreadsheets and will be made available on reasonable request.
